# Cyclin‐dependent kinase activity enhances phosphatidylcholine biosynthesis in Arabidopsis by repressing phosphatidic acid phosphohydrolase activity

**DOI:** 10.1111/tpj.13321

**Published:** 2016-12-01

**Authors:** Christian P. Craddock, Nicolette Adams, Johan T.M. Kroon, Fiona M. Bryant, Patrick J. Hussey, Smita Kurup, Peter J. Eastmond

**Affiliations:** ^1^ School of Life SciencesUniversity of WarwickCoventryCV4 7ALUK; ^2^ School of Biological and Biomedical SciencesDurham UniversityDurhamDH1 3LEUK; ^3^ Department of Plant Biology and Crop ScienceRothamsted ResearchHarpendenHertfordshireAL5 2JQUK; ^4^ Present address: Center for Plant Cell BiologyDepartment of Botany and Plant SciencesUniversity of CaliforniaRiverside92521USA; ^5^ Present address: Centre for Proteomic and Genomic ResearchUpper LevelSt Peter's MallCorner Anzio and Main Road ObservatoryCape Town7925South Africa; ^6^ Present address: School of Biological and Biomedical SciencesDurham UniversityDurhamDH1 3LEUK

**Keywords:** *Arabidopsis thaliana*, phosphatidylcholine, phospholipid biosynthesis, membrane biogenesis, phosphatidic acid phosphohydrolase, phosphorylation, cell cycle, cyclin‐dependent kinase

## Abstract

Coordination of endomembrane biogenesis with cell cycle progression is considered to be important in maintaining cell function during growth and development. We previously showed that the disruption of PHOSPHATIDIC ACID PHOSPHOHYDROLASE (PAH) activity in *Arabidopsis thaliana* stimulates biosynthesis of the major phospholipid phosphatidylcholine (PC) and causes expansion of the endoplasmic reticulum. Here we show that PC biosynthesis is repressed by disruption of the core cell cycle regulator CYCLIN‐DEPENDENT KINASE A;1 (CDKA;1) and that this repression is reliant on PAH. Furthermore, we show that cyclin‐dependent kinases (CDKs) phosphorylate PAH1 at serine 162, which reduces both its activity and membrane association. Expression of a CDK‐insensitive version of PAH1 with a serine 162 to alanine substitution represses PC biosynthesis and also reduces the rate of cell division in early leaf development. Together our findings reveal a physiologically important mechanism that couples the rate of phospholipid biosynthesis and endomembrane biogenesis to cell cycle progression in Arabidopsis.

## Significance Statement

Coordinating endomembrane biogenesis as cells progress thorough the cell cycle is important. Here we uncover a CYCLIN‐DEPENDENT KINASE A;1 regulatory mechanism that couples the rate of phospholipid biosynthesis and endomembrane biogenesis.

## Introduction

Plant growth and development are primarily driven by the production of new cells (Polyn *et al*., 2015). In addition to DNA replication, cell proliferation requires an approximate doubling of core structural components such as the endomembrane system prior to each division (Jackowski, 1994, 1996). Phospholipids are the main building blocks of the plant endomembrane system (Ohlrogge and Browse, 1995) and hence their biosynthesis must occur in conjunction with cell cycle progression. Temporal changes in phospholipid metabolism over the course of the cell cycle have been well documented in mammals, fungi, algae, dinoflagellates and bacteria (Janero and Barrnett, 1981; Joseleau‐Petit *et al*., 1984; Knacker *et al*., 1985; Jackowski, 1994; Saitoh *et al*., 1996; Kwok and Wong, 2005). Similar changes must also take place in plants but surprisingly they have yet to be described, possibly because of difficulties in generating highly synchronized plant cell cultures (Kwok and Wong, 2005). Furthermore, there is currently no mechanistic understanding of how phospholipid biosynthesis is coupled to the cell cycle in plants.

The cell cycle is governed by conserved molecular machinery in which the core components are serine/threonine kinases, known as cyclin‐dependent kinases (CDKs) (Inzé and De Veylder, 2006; Gutierrez, 2009; Polyn *et al*., 2015). The link between CDK activity and phospholipid biosynthesis is perhaps best understood in the budding yeast *Saccharomyces cerevisiae* where the enzyme Mg^2+^‐dependent phosphatidic acid phosphohydrolase (Pah1p) is a direct target for multisite phosphorylation by both cell division cycle 28 (Cdc28p) and phosphate metabolism 85 (Pho85p) (Santos‐Rosa *et al*., 2005; Choi *et al*., 2011, 2012). Together Cdc28p and Pho85p modulate a range of cellular processes in response to environmental stimuli (Enserink and Kolodner, 2010). Pah1p catalyses the conversion of phosphatidic acid (PA) to diacylglycerol (DAG), which is a key step controlling the partitioning of carbon flux between membrane and storage lipid biosynthesis in *S. cerevisiae* (Carman and Henry, 2007). Pah1p is an amphitropic enzyme that switches between a phosphorylated soluble inactive form and a dephosphorylated membrane‐bound active form (Choi *et al*., 2011). Pah1 is dephosphorylated by the nuclear envelope morphology 1–sporulation 7 (Nem1p–Spo7p) protein phosphatase complex at the nuclear‐endoplasmic reticulum (ER) membrane allowing the protein to associate with the membrane via a short N‐terminal amphipathic helix (Karanasios *et al*., 2010; Choi *et al*., 2011).

Pah1p membrane disassociation and inactivation resulting from multisite phosphorylation by Cdc28p and Pho85p, leads to elevated levels of the enzymes’ substrate (PA), which is the precursor for the cytidine diphosphate‐diacylglycerol (CDP‐DAG) pathway. This pathway is able to synthesise all phospholipid classes in *S. cerevisiae* (Carman and Henry, 2007). Accumulation of PA also enhances the expression of a suite of phospholipid biosynthetic genes and causes dramatic expansion of the nuclear‐ER membrane (Santos‐Rosa *et al*., 2005; Carman and Henry, 2007). The genes that are induced form part of the inositol‐responsive circuit in *S. cerevisiae* (Carman and Henry, 2007). Expression of these genes is induced by the inositol requiring 2–inositol requiring 4 (Ino2p‐Ino4p) transcription factor complex, but is blocked by interaction of the repressor protein overproducer of inositol 1 (Opi1p) with Ino2p (Carman and Henry, 2007). Accumulation of PA relieves Opi1p‐mediated repression of gene expression because it ties Opi1p to a protein called suppressor of choline sensitivity 2 at the nuclear–ER membrane (Loewen *et al*., 2004).

Orthologues of *S. cerevisiae* Pah1 are also present in higher eukaryotes where they are often termed lipins (Carman and Henry, 2007). In both animals and plants, it has been shown that disruption of lipins can enhance PA content, trigger changes in nuclear and/or ER membrane morphology (Golden *et al*., 2009; Gorjánácz and Mattaj, 2009; Eastmond *et al*., 2010; Ugrankar *et al*., 2011; Han *et al*., 2012; Mall *et al*., 2012; Bahmanyar *et al*., 2014) and alter gene expression (Finck *et al*., 2006; Donkor *et al*., 2009; Eastmond *et al*., 2010; Peterson *et al*., 2011; Craddock *et al*., 2015). In the case of *Caenorhabditis elegans,* although defects in nuclear and ER morphology are accompanied by a perturbation in phospholipid composition, total phospholipid content does not increase significantly (Bahmanyar *et al*., 2014). *Arabidopsis thaliana* contains two Pah1p orthologs called PAH1 and PAH2, which appear to be expressed in all tissues (Nakamura *et al*., 2009; Eastmond *et al*., 2010). Analysis of a double mutant shows that extra‐plastidial phospholipid content almost doubles and both the rate of phosphatidylcholine (PC) biosynthesis and turnover increases markedly (Eastmond *et al*., 2010; Wang *et al*., 2014; Craddock *et al*., 2015). In *A. thaliana* there are also gross changes in ER morphology, but unlike *C. elegans* there is no obvious defect in the nucleus (Eastmond *et al*., 2010; Bahmanyar *et al*., 2014). These data suggest that plants may use PAHs as a hub in the regulatory network governing phospholipid biosynthesis and endomembrane biogenesis (Eastmond *et al*., 2010; Craddock *et al*., 2015).

The mechanism by which PAH inactivation enhances phospholipid (and in particular PC) production in *A. thaliana* differs markedly from that of *S. cerevisiae*. This is because the inositol‐responsive circuit found in *S. cerevisiae* (Carman and Henry, 2007) is not conserved in higher eukaryotes and *A. thaliana* also does not use PA to synthesise PC via the CDP‐DAG pathway, because it cannot directly methylate phosphatidylethanolamine (PE) (Keogh *et al*., 2009). Instead it converts ethanolamine to choline by methylation mainly at the phospho‐base level and uses the nucleotide (also known as Kennedy) pathway to assemble PC from CDP‐choline and DAG (Keogh *et al*., 2009). Because DAG is the product of the reaction catalysed by PAH it is counterintuitive that disruption of this enzyme should lead to an increase in PC biosynthesis (Eastmond *et al*., 2010). However, characterisation of the *pah1 pah2* mutant shows that *A. thaliana* possesses additional routes to synthesise DAG (Nakamura *et al*., 2009; Eastmond *et al*., 2010) and isotopic labelling experiments indicate that the rate of PC biosynthesis is more limited by the availability of CDP‐choline, supplied by the enzyme cytidine 5′‐triphosphate: phosphocholine cytidylyltransferase (CCT) (Kinney *et al*., 1987; Eastmond *et al*., 2010). Analysis of PC biosynthesis in the *pah1 pah2* mutant suggests that enhanced metabolic flux through the pathway is mainly a result of increased CCT activity (Eastmond *et al*., 2010; Craddock *et al*., 2015). In many eukaryotes CCT activity is determined by local membrane lipid composition with a relative enrichment of anionic lipids such as PA and its CDP‐DAG pathway derivatives phosphatidylinositol (PI) and phosphatidylglycerol (PG) acting as a stimulant (Cornell and Northwood, 2000; Jackowski and Fagone, 2005). Arabidopsis CCT1 activity can also be stimulated by anionic lipids and expression of a lipid‐insensitive version partially mimics the *pah1 pah2* phenotype by stimulating both PC biosynthesis and ER proliferation (Craddock *et al*., 2015). These data suggest that in the *pah1 pah2* mutant, PC biosynthesis increases to compensate for an imbalance in membrane lipid composition (Craddock *et al*., 2015). Interestingly, Szymanski *et al*. (2014) also reported that disruption of *CDP‐DAG SYNTHASE2* not only reduces PI and PG levels in *A. thaliana,* but also those of PC and PE.

Regardless of the precise mechanism of action, PAH activity has the potential to modulate phospholipid biosynthesis in *A. thaliana* (Eastmond *et al*., 2010; Wang *et al*., 2014; Craddock *et al*., 2015). This study aimed to investigate how PAH activity is governed, and particularly whether it might be coupled to cell cycle progression via the action of CDKs, as has been shown in *S. cerevisiae* (Santos‐Rosa *et al*., 2005; Karanasios *et al*., 2010; Choi *et al*., 2011, 2012). *A. thaliana* contains twelve CDKs that are classified into six groups, but only class A and class B CDKs have been implicated in core cell cycle regulation in plants (Inzé and De Veylder, 2006; Gutierrez, 2009). Among *A. thaliana* CDKs, *S. cerevisiae* Cdc28p and Pho85p share the greatest sequence similarity with CDKA;1, which is the only class A CDK present in *A. thaliana* and is distinguished by the conserved PSTAIRE cyclin‐binding motif (Nowack *et al*., 2012) that is also found in both Cdc28p and Pho85p. *CDKA;1* is expressed throughout the cell cycle (Segers *et al*., 1996), both in tissues with active cell division and also those with proliferative competence (Martinez *et al*., 1992). CDKA;1 can complement the *S. cerevisiae cdc28Δ* mutant suggesting that it functions in both G1 to S and G2 to M phase transitions (Ferreira *et al*., 1991). Mutant analysis in *A. thaliana* has confirmed that CDKA;1 is necessary for entry into S phase and thus for both mitotic cell division and endoreduplication (Nowack *et al*., 2012). We therefore focused our investigation on whether CDKA;1 might play a role in governing PAH activity in *A. thaliana*.

## Results

### Disruption of CDKA;1 enhances PAH activity and suppresses PC biosynthesis

**Figure 1:**
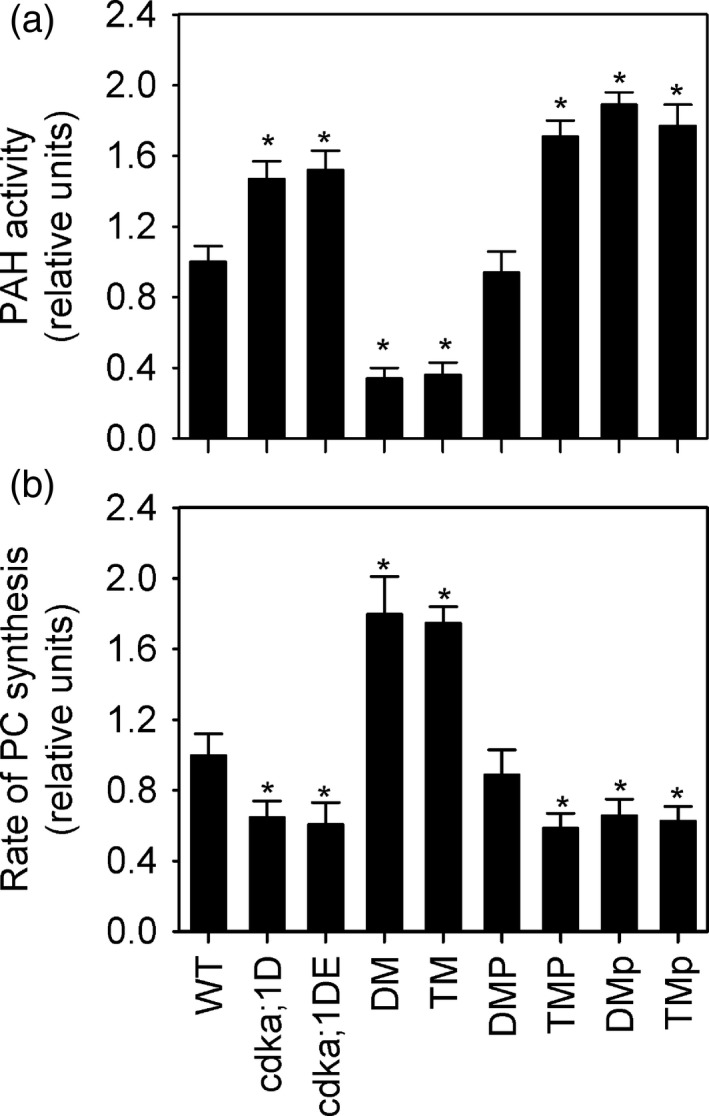
Effect of CDKA;1 and PAH disruption on PAH activity and PC biosynthesis. (a, b) Total PAH activity (a) and rate of [*methyl*‐^14^C]choline incorporation into PC (b) were measured in leaf tissue of wild type (WT), *cdka;1D* and *cdka;1DE, pah1 pah2* (DM), *pah1 pah2 cdka;1D* (TM), *pah1 pah2* (DMP) or *pah1 pah2 cdka;1D* (TMP) expressing PAH1‐HA under the CaMV 35S promoter and *pah1 pah2* (DMp) and *pah1 pah2 cdka;1D* (TMp) expressing PAH1^S162A^‐HA under the CaMV 35S promoter. Values are the mean ± SE of measurements on four separate pools of leaf material from each genotype. Asterisks denote a statistically significant difference from wild type (WT) (*P* < 0.05).

In *Arabidopsis thaliana* it is possible to obtain null mutants in *CDKA;1* (Nowack *et al*., 2012), but their growth is so severely retarded that biochemical analysis is problematic. However, weaker alleles of *cdka;1* (referred to here as *cdka;1D* and *cdka;1DE*) have been created by transforming *cdka;1* plants with construct expressing phosphomimicry versions of CDKA;1 that have greatly reduced kinase activity (Dissmeyer *et al*., 2007, 2009). The *cdka;1D* line expresses a copy of CDKA;1 with a T161D substitution and the *cdka;1DE* line a copy with T14D and Y15E substitutions (Dissmeyer *et al*., 2007, 2009). To determine whether CDKA;1 function effects the activity of PAH in *A. thaliana* we performed enzyme assays (Eastmond *et al*., 2010) on extracts from developing rosette leaves of wild type, *cdka;1D* and *cdka;1DE* (Figure 1). PAH activity was increased by ~50% in both *cdka;1D* and *cdka;1DE* on a per unit fresh weight basis (Figure 1a). To investigate whether CDKA;1 function also effects phospholipid metabolism we measured the rate of PC biosynthesis in the leaves by monitoring the incorporation of [*methyl*‐^14^C]choline into lipids (Eastmond *et al*., 2010). The rate of biosynthesis was ~30% lower in *cdka;1D* and *cdka;1DE* plants than in wild type when expressed on a per unit fresh weight basis (Figure 1b). The total PC content of *cdka;1D* and *cdka;1DE* leaves was also lower (Table S1). Since a reduction in PAH activity leads to stimulation of PC biosynthesis and ER biogenesis in *A. thaliana* (Eastmond *et al*., 2010; Wang *et al*., 2014; Craddock *et al*., 2015), it is logical that the increase observed in *cdka;1D* and *cdka;1DE* plants may be responsible for the suppression of the pathway.

### Suppression of PC biosynthesis by CDKA;1 is dependent on PAH activity

To investigate whether the elevated PAH activity in *cdka;1D* leaves is derived from PAH1 and/or PAH2 and whether this increase is responsible for the suppression of PC biosynthesis we created a *pah1 pah2 cdka;1D* triple mutant (Figure 1). The increase in PAH activity detected in *cdka;1D* leaves was suppressed in *pah1 pah2 cdka;1D* (Figure 1(a)) and therefore is attributable to the PAH1 and/or PAH2 proteins, and not to other lipid phosphatases that are responsible for the remaining PAH activity present in *pah1 pah2* (Nakamura *et al*., 2009; Eastmond *et al*., 2010). The rate of incorporation of [*methyl*‐^14^C]choline into lipids in *cdka;1D* was also recovered in the *pah1 pah2* background to a rate that is substantially higher than wild type (Figure 1b). Leaf area, cell number and cell size of *pah1 pah2 cdka;1D* plants were not significantly different (*P* > 0.05) to those of *cdka;1D* (Table S2) suggesting that the growth defects caused by CDKA;1 disruption (Dissmeyer *et al*., 2007) cannot be rescued by relieving the repression of phospholipid synthesis. Considered together these data suggest that CDKA:1 suppresses PAH activity and enhances PC biosynthesis, and that CDKA;1 exerts its effect on PC production in *A. thaliana* through PAH.

### PAH1 is sufficient to mediate the effect of CDKA;1 on PC biosynthesis

**Figure 2:**
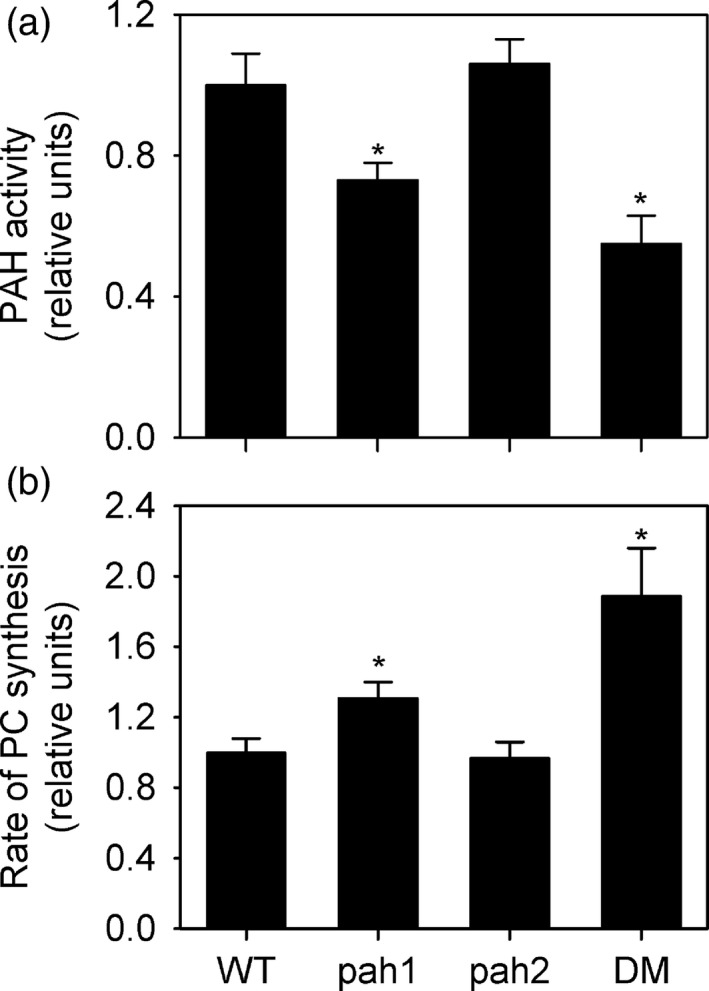
Effect of PAH1 and PAH2 disruption on PAH activity and PC biosynthesis single mutants. (a, b) Total PAH activity (a) and rate of [*methyl*‐^14^C]choline incorporation into PC (b) and were measured in 4‐week‐old leaf tissue of wild type (WT), *pah1, pah2* and *pah1 pah2* (DM). Values are the mean ± SE of measurements on four separate pools of leaf material from each genotype. Asterisks denote a statistically significant difference from WT (*P* < 0.05).

*A. thaliana* contains two *PAH* genes with overlapping expression patterns (Eastmond *et al*., 2010). Single *pah1* and *pah2* mutants have no obvious morphological phenotypes (Eastmond *et al*., 2010). However, the *pah1* mutant does exhibit a significant (*P* < 0.05) reduction in PAH activity and elevated rate of PC biosynthesis (Figure 2). PAH1 is therefore the predominant isoform in this tissue. We have previously shown that constitutive expression of hemagglutinin (HA) tagged PAH1 under the control of the CaMV 35S promoter can complement the *pah1 pah2* mutant (Craddock *et al*., 2015). To determine whether CDKA;1 can exert its effect on PC biosynthesis through PAH1 we introduced the same Pro35S:PAH1‐HA T‐DNA insertion event into the *pah1 pah2 cdka;1D* background via crossing (Figure 1). The increase in PAH activity detected in *cdka;1D* leaves, but suppressed in *pah1 pah2 cdka;1D,* was rescued by Pro35S:PAH1‐HA (Figure 1a). Furthermore, the rate of incorporation of [*methyl*‐^14^C]choline into lipids that was supressed in *cdka;1D,* but recovered in *pah1 pah2 cdka;1D,* was again suppressed by Pro35S:PAH1‐HA (Figure 1b). These data suggest that the activity of PAH1 is supressed by CDKA;1 and that, in the absence of PAH2, this suppression is sufficient to stimulate PC biosynthesis.

### CDK‐cyclin complexes can phosphorylate PAH1 *in vitro* and inhibit its activity

**Figure 3:**
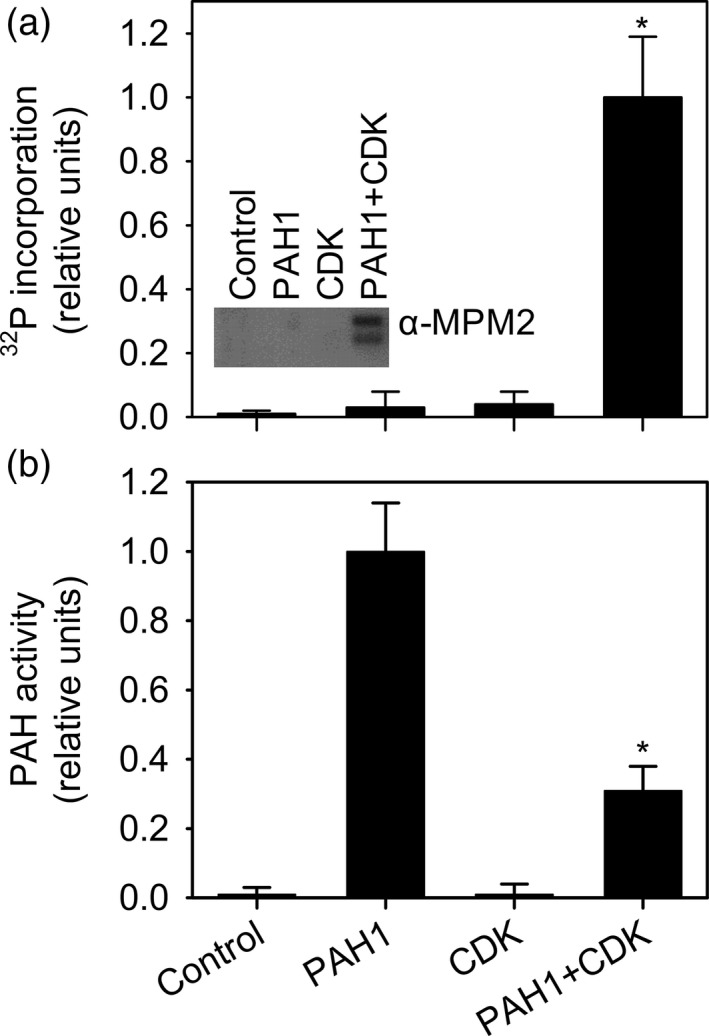
Phosphorylation of recombinant His6‐PAH1 by purified p13^Suc1^‐bound CDK‐cyclin complexes. (a) CDK‐dependent incorporation of ^32^P from [γ‐^32^P]ATP into PAH1 protein. Inset is an immunoblot probed with anti‐pS/pT–P (α‐MPM2) antibodies. Two bands are present here because purified His6‐PAH1 is partially degraded (Eastmond *et al*., 2010). (b) Inhibition of PAH1 activity by incubation with ATP and CDK‐cyclin complexes. Values are the mean ± SE of measurements on four separate incubations. Asterisks denote a statistically significant difference from PAH1 alone (*P* < 0.05).

To investigate whether PAH1 is a substrate for CDK‐cyclin complexes, histidine x 6‐tagged PAH1 was expressed in *Escherichia coli* and affinity purified as previously described (Eastmond *et al*., 2010). *In vitro* phosphorylation assays were carried out on the recombinant protein using p13^Suc1^‐bound CDK‐cyclin complexes isolated from *A. thaliana* flower bud extracts (Dissmeyer *et al*., 2007) and [γ‐^32^P]ATP (Figure 3). Kinase activity was detected, which required the presence of both the protein substrate (His6‐PAH1) and the CDKA;1‐containing complex (Figure 3a). CDKs commonly phosphorylate the consensus site S/T‐P in target proteins (Nowack *et al*., 2012) and anti‐pS/pT‐P antibodies (α‐MPM2) have previously been used to detect Cdc28p and Pho85p dependent phosphorylation of Pah1p (Santos‐Rosa *et al*., 2005; Choi *et al*., 2011, 2012). Using α‐MPM2 antibodies we were also able to detect CDK‐dependent phosphorylation of His6‐PAH1 by immunoblotting (Figure 3a). To screen directly for CDK‐dependent phosphorylation sites the products of unlabelled reactions were separated by gel electrophoresis. His6‐PAH1 was then subjected to tryptic digestion and the resulting peptides were separated via liquid chromatography and analysed by tandem mass spectrometry (LC‐MS/MS). As shown previously (Eastmond *et al*., 2010), diagnostic mass fingerprints could be identified for 33 peptides, representing 43% coverage of the PAH1 protein. In addition to these peptides, a single phosphorylated peptide was also detected (FYDFQDDPP[pS]PTSEYGSAR) with a modification that corresponds to serine 162 in the intact native protein (Figure S1). Evidence from multiple proteomics studies contained in the phosphat 4.0 database (Durek *et al*., 2010) suggests that this site is also phosphorylated *in vivo* (Ito *et al*., 2009; Jones *et al*., 2009; Nakagami *et al*., 2010; Reiland *et al*., 2011; Wang *et al*., 2013; Zhang *et al*., 2013). To investigate whether CDK‐dependent phosphorylation of His6‐PAH affects its activity, enzyme assays were also performed on the reaction products. CDK treatment led to a ~65% reduction in PAH activity (Figure 3b). These data suggest that PAH1 can be phosphorylated and inactivated by CDKs *in vitro* and that S162 is a target.

### CDKA;1 is required for *in vivo* PAH1 phosphorylation

**Figure 4:**
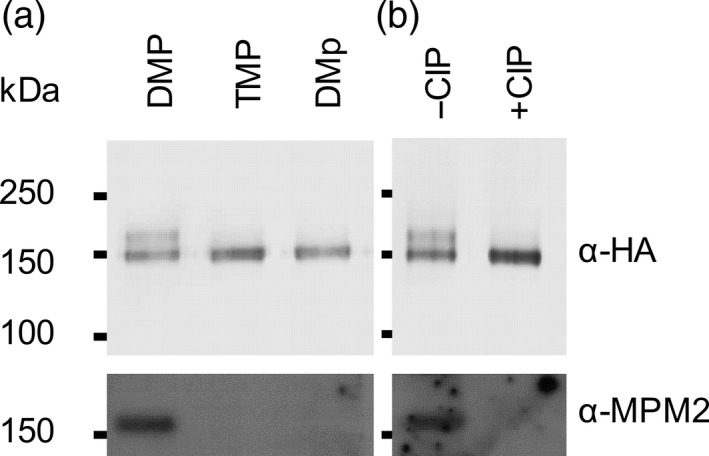
Effect of CDKA;1 disruption and S162A substitution on *in vivo* phosphorylation state of PAH1. Immunoblots of immunoprecipitated PAH1‐HA separated on a 7.5% SDS‐PAGE gel and probed with α‐HA or anti‐pS/pT–P (α‐MPM2) antibodies. (a) Immunoprecipitate from *pah1 pah2 Pro35S:PAH1‐HA* (DMP), *pah1 pah2 cdka;1D Pro35S:PAH1‐HA* (TMP) and *pah1 pah2 Pro35S:PAH1* ^*S162A*^ *‐HA* (DMp). (b) Immunoprecipitate from *pah1 pah2 Pro35S:PAH1‐HA* before and after treatment with Calf intestinal alkaline phosphatase (CIP) solution.

To investigate whether CDKA;1 activity effects the *in vivo* phosphorylation state of PAH1, and whether S162 plays a role in this, we created a dephosphorylated version of PAH1‐HA containing a S162A substitution (Dissmeyer and Schnittger, 2011) and expressed it in *pah1 pah2* under the control of the CaMV 35S promoter. In *S. cerevisiae,* phosphorylation of Pah1p by Cdc28p and Pho85p reduces its electrophoretic mobility (Santos‐Rosa *et al*., 2005; Choi *et al*., 2011, 2012). Therefore, we immunoprecipitated PAH1‐HA and PAH1^S162A^‐HA from *pah1 pah2* leaf extracts and PAH1‐HA from *pah1 pah2 cdka;1D* leaf extract and performed gel electrophoresis followed by immunoblotting (Figure 4). From the *pah1 pah2* genetic background α‐HA antibodies detected PAH1‐HA bands of differing mobility, whereas from *pah1 pah2 cdka;1D* a single dominant band was detected with the same mobility as the lowest band present in *pah1 pah2* (Figure 4a). From *pah1 pah2* expressing PAH1^S162A^‐HA the single lower band was also dominant (Figure 4a). Using α‐MPM2 antibodies we were also able to detected a band in immunoprecipitates from *pah1 pah2* expressing PAH1‐HA, but were unable to from *pah1 pah2 cdka;1D* expressing PAH1‐HA or *pah1 pah2* expressing PAH1^S162A^‐HA (Figure 4). To confirm that the shift in mobility and signal detected by α‐MPM2 were both due to phosphorylation of PAH1‐HA, immunoprecipitates were treated with Calf intestinal alkaline phosphatase (CIP) prior to immunodetection (Figure 4b). Together these data suggest that PAH1 is phosphorylated at S162 in a CDKA;1‐dependent manner.

### Phosphorylation of PAH1 is necessary for stimulation of PC biosynthesis

To investigate whether the phosphorylation state of PAH1 at S162 plays a role in governing PAH activity and PC biosynthesis in *A. thaliana,* we introduced 35S:PAH1^S162A^‐HA into the *pah1 pah2 cdka;1D* background (Figure 1). PAH activity in *pah1 pah2* leaves expressing PAH1^S162A^‐HA was significantly higher (*P* < 0.05) than in those expressing the wild type form of PAH1‐HA (Figure 1a). This was in spite of the fact that the abundance of protein is very similar (Figure 4a). By contrast, expression of PAH1^S162A^‐HA and PAH1‐HA from the same transformation events in *pah1 pah2 cdka;1D* plants resulted in no significant difference (*P* > 0.05) in PAH activity (Figure 1a). The rate of incorporation of [*methyl*‐^14^C]choline into PC in *pah1 pah2* leaves expressing PAH1^S161A^‐HA was also significantly lower (*P* < 0.05) than in *pah1 pah2* leaves expressing PAH1‐HA, but there was no significant difference (*P* > 0.05) in *pah1 pah2 cdka;1D* leaves (Figure 1b). These data suggest that CDKA;1‐dependent phosphorylation of PAH1 at S162 suppresses PAH activity and through this action enhances PC biosynthesis.

### Phosphorylation of PAH1 effects membrane association as well as activity

**Figure 5:**
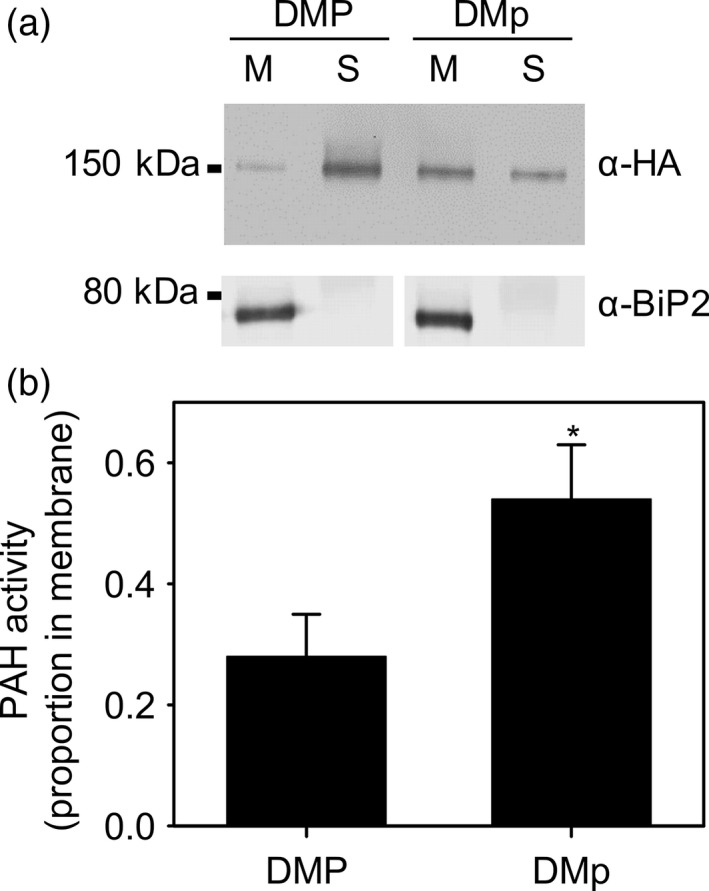
Effect of PAH1 non‐phosphorylated S162A substitution on subcellular localization. (a) Immunoblot of membrane‐bound (M) and soluble (S) protein fractions separated on a 12% SDS‐PAGE gel. (b) Proportion of PAH activity present in the membrane versus soluble fraction. DMP is *pah1 pah2* expressing PAH1‐HA and DMp is *pah1 pah2* expressing PAH1^S162A^‐HA. The ER marker protein BiP2 is shown in (a) as a control. (b) In (b) values are the mean ± SE of measurements on three separate fractionation experiments and the proportion of phospho*enol*pyruvate carboxylase (cytosolic marker) activity in the membrane fraction was <0.01 in both DMP and DMp. The asterisk denotes a statistically significant difference from DMP (*P* < 0.05).

The phosphorylation status of Pah1p determines the protein's localization, as well as its activity in *S. cerevisiae*. The N‐terminus of Pah1 contains an amphipathic helix responsible for membrane binding and phosphorylation by Cdc28p and Pho85p causes a conformational change that disassociates the protein from the membrane (Karanasios *et al*., 2010; Choi *et al*., 2011, 2012). In *A. thaliana,* both C‐ and N‐terminally tagged PAH1 and PAH2 are predominantly soluble proteins when expressed under the CaMV 35S promoter (Nakamura *et al*., 2009; Eastmond *et al*., 2010). However, sequence analysis (Gautier *et al*., 2008) suggests that the amphipathic helix found at the N‐terminus in *S. cerevisiae* Pah1p might also be a conserved feature in PAH1 and PAH2 (Figure S2). To study whether phosphorylation of PAH1 affects the localization of the protein we used immunoblotting to monitor PAH1‐HA protein distribution in subcellular fractions of *pah1 pah2* leaf extracts expressing either PAH1‐HA or PAH1^S162A^‐HA (Figure 5). In the case of PAH1‐HA, the majority of protein was detected in the soluble (150 000 g supernatant) fraction and only traces were detected in the microsomal membrane fraction (150 000 g pellet) (Figure 5a). This finding is consistent with those of Nakamura *et al*. (2009) who previously performed fractionation experiments on extracts from leaves expressing PAH1 tagged at the C‐terminus with green fluorescent protein (GFP). However, in extracts from *pah1 pah2* leaves expressing PAH1^S162A^‐HA, a more even distribution of protein was detected between the microsomal membrane fraction and the soluble fraction (Figure 5a). The membrane fraction from *pah1 pah2* leaves already contains PAH activity, which must be derived from other lipid phosphatases (Nakamura *et al*., 2009). However, enzyme assays performed on the different fractions from *pah1 pah2* leaves expressing PAH1‐HA or PAH1^S162A^‐HA show that the proportion of PAH activity in the membrane fraction is significantly (*P* < 0.05) enhanced in the case of PAH1^S162A^‐HA (Figure 5b). Together these data support the hypothesis that phosphorylation of PAH1 by CDKA;1 reduces membrane association as well as activity.

### Phosphorylation of PAH1 is required for normal rates of cell division

**Figure 6:**
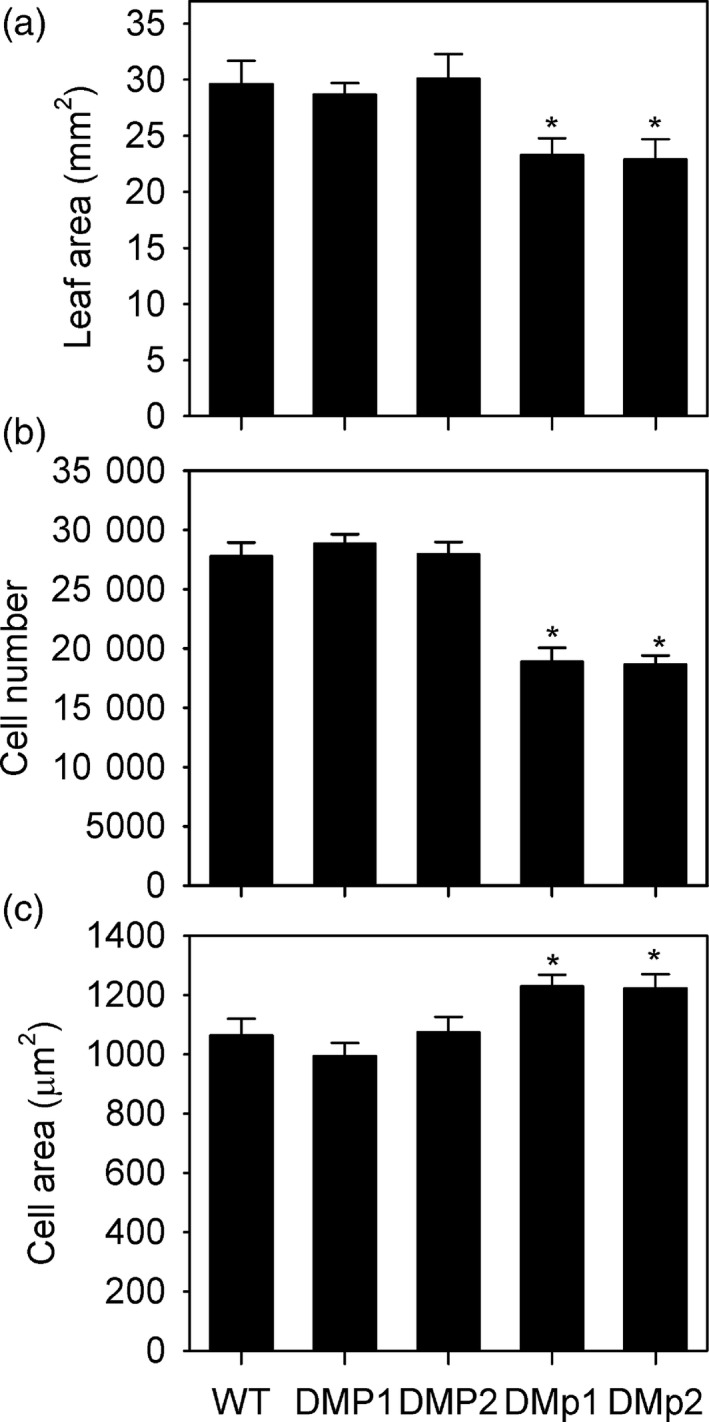
Effect of PAH1 non‐phosphorylated S162A substitution on mature leaves. A comparison of leaf area (a) cell number (b) and cell area (c) for the first true leaf pair of four‐week‐old wild type (WT) plants and *pah1 pah2* plants expressing PAH1‐HA (DMP) or PAH1^S162A^‐HA (DMp). Two independent transformation events (1 and 2) were analysed for each construct. Values are the mean ± standard error (SE) of measurements on leaves from three to six plants of each genotype. Asterisks denote a statistically significant difference from WT (*P* < 0.05).

**Figure 7:**
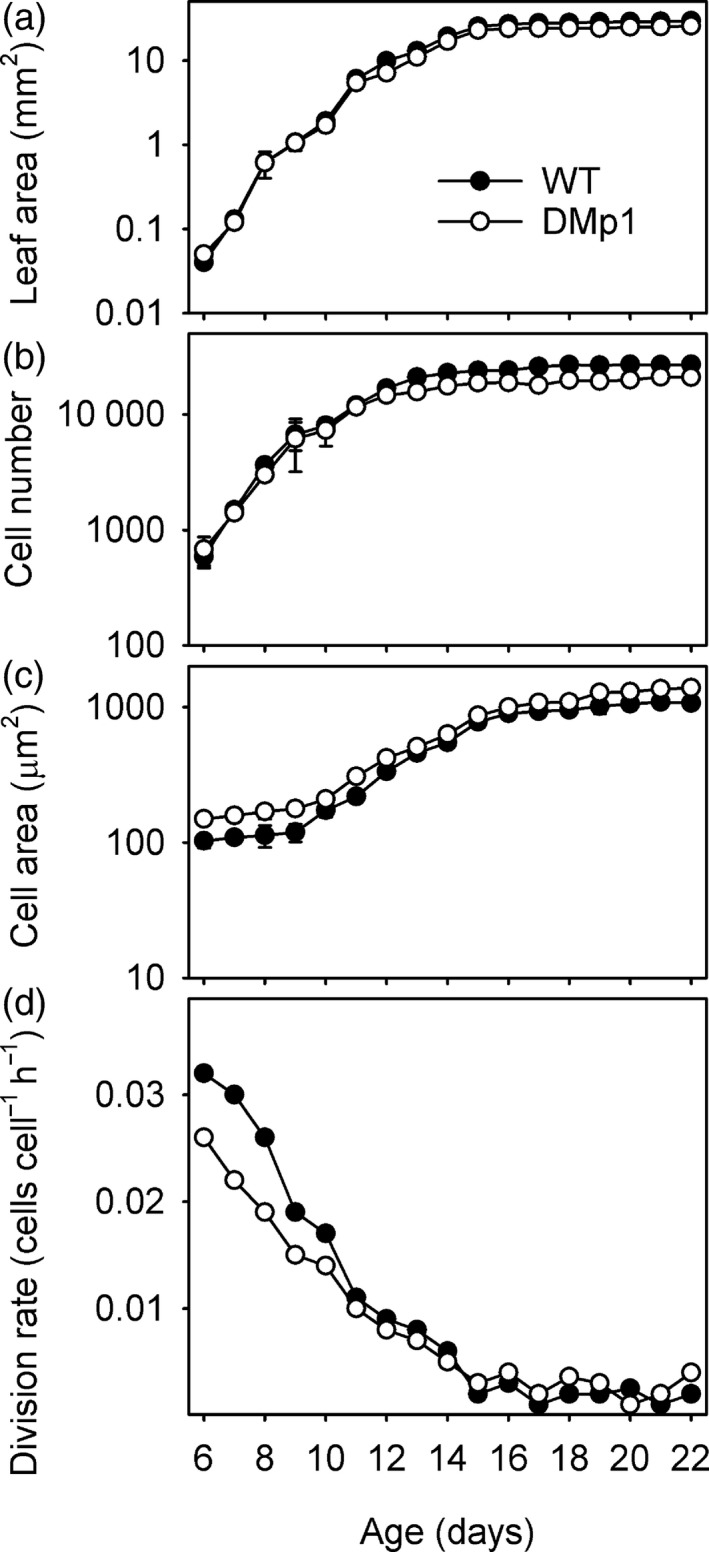
Kinematic analysis of leaves expressing PAH1^S162A^. Leaf area (a) cell number (b) cell area (c) and cell division rate (d) for the first leaf pair of wild type (WT) plants and *pah1 pah2* plants expressing PAH1^S162A^‐HA (DMp). Values are the mean ± standard error (SE) of measurements on the first true leaf pair from three to eight plants of each genotype.

We have shown that expressing the dephosphorylated version of PAH1 in *A. thaliana* results in enhanced PAH activity and membrane residency in leaves and that consequently the rate of PC biosynthesis is repressed (Figures 1 and 5). To investigate whether this has an impact on leaf growth and development, wild type (Col‐0) plants and *pah1 pah2* plants expressing either PAH1‐HA or PAH1^S162A^‐HA were grown alongside each other and their phenotype compared (Figure 6). Wild type plants and *pah1 pah2* plants expressing PAH1‐HA were indistinguishable at rosette stage. However, *pah1 pah2* plants expressing PAH1^S162A^‐HA exhibited a small but significant reduction (*P* < 0.05) in the area of mature rosette leaves (Figure 6a). The effect of PAH1^S162A^‐HA expression on leaf growth was investigated in more detail. Microscopic analysis of the abaxial surface of mature leaves showed that the number of epidermal cells was significantly reduced (*P* < 0.05) in *pah1 pah2* plants expressing PAH1^S162A^‐HA, as compared to either wild type or *pah1 pah2* plants expressing PAH1‐HA (Figure 6b). By contrast, the average area of each cell was significantly (*P* < 0.05) increased (Figure 6c). These data suggest a defect in the rate (or duration) of cell division (De Veylder *et al*., 2001). To study this further, we performed kinematic analysis of cell division and expansion in the first true leaf pair of wild type (WT) and *pah1 pah2* expressing PAH1^S162A^‐HA (DMp1). In both genotypes, leaf area and abaxial epidermal cell number increased exponentially until around days 10 to 12 and then the rates decreased (Figure 7a,b). Average cell area also remained stable in both genotypes until day 10 and then began to increase exponentially (Figure 7c). In both genotypes the calculated rate of cell division was greatest at the earliest time point (day 6) and declined (Figure 7d), as cell expansion accelerated (Figure 7c). These data suggest that the period of most rapid cell division is not substantially altered in DMp1. However, within this phase (day 6 to 10) the rate was consistently lower in DMp1 than in WT (Figure 7d). These data suggest that phosphorylation of PAH1 at S162 is required for normal rates of cell division (De Veylder *et al*., 2001). Since we have shown that phosphorylation of S162 in PAH1 is reduced in leaves of the *cdka;1D* mutant, our data support the hypothesis that CDK‐specific suppression of PAH1 catalytic function drives sufficient endomembrane production to allow normal rates of mitotic cell division during leaf growth and development in *A. thaliana*.

## Discussion

In this study, we have provided evidence that the core cell cycle regulator CDKA;1 (Nowack *et al*., 2012) stimulates the rate of PC biosynthesis in *Arabidopsis thaliana* by phosphorylating the enzyme PAH. This post‐translational modification reduces the catalytic activity of PAH and also impairs protein association with the membrane, where its substrate resides. We have previously shown that suppression of PAH activity enhances phospholipid content and causes ER proliferation (Eastmond *et al*., 2010). The mechanism of PAH action is yet to be fully understood, but the increase in the rate of PC biosynthesis would appear to be caused by activation of the rate‐limiting enzyme CCT (Craddock *et al*., 2015). Taken together our data provide a mechanistic link to explain how phospholipid biosynthesis in plant cells is coordinated with cell cycle progression. We have also shown that expression of a dephosphorylated form of PAH1 uncouples PC biosynthesis from CDKA;1 and reduces the rate of mitotic cell division in the leaf. This suggests that CDKA;1 phosphorylation of PAH1 plays a physiologically important role in plant growth and development. Defects in the biosynthesis of fatty acids and lipids have been shown to impair cell division in many eukaryotic systems (Cornell *et al*., 1977; Tercé *et al*., 1994; Saitoh *et al*., 1996; Al‐Feel *et al*., 2003; Kwok and Wong, 2005; Bach *et al*., 2011; Atilla‐Gokcumen *et al*., 2014). At the most basic level coupling between the two processes is considered to be important because membranes are a basic building block of the cell and their constituent phospholipids must increase in mass prior to each division (Jackowski, 1994, 1996). There is evidence that specific molecular species of membrane lipids also play specialised roles in cell division (Al‐Feel *et al*., 2003; Bach *et al*., 2011; Atilla‐Gokcumen *et al*., 2014) and may even act as signals to govern cell cycle progression (Koeberle *et al*., 2013; Chauhan *et al*., 2015).

It is accepted that phospholipid metabolism changes dynamically over the course of the cell cycle with the pattern varying depending on species (Jackowski, 1994, 1996). For yeasts such as *S. cerevisiae* and *Schizosaccharomyces pombe* that undergo closed cell division there is a burst of phospholipid biosynthesis upon entry to mitosis in order to support the rapid expansion of the nuclear envelope (Santos‐Rosa *et al*., 2005; Makarova *et al*., 2016). In accordance with this, CDK‐dependent phosphorylation of both *S. cerevisiae* Pah1p and its *S. pombe* ortholog Ned1 (nuclear elongation and deformation protein 1) increases upon G2 to M transition (Santos‐Rosa *et al*., 2005; Makarova *et al*., 2016). By contrast in mammalian cells, which undergo open cell division, net accumulation of phospholipids is generally associated with cell (and nuclear) expansion during S phase (Cornell *et al*., 1977; Jackowski, 1994). Interestingly in the yeast *S. japonicas,* which also uses open cell division, Ned1 is not hyperphosphorylated during G2 to M transition despite being a CDK substrate (Makarova *et al*., 2016). Unfortunately, it is not known how phospholipid metabolism varies over the course of the cell cycle in plants (Kwok and Wong, 2005). However, given that plants employ open cell division, it is possible that phospholipid accumulation might also be associated with S phase.

Our data suggest that PAH1 phosphorylation by CDKA;1 at S162 is important for enzyme function, but it remains possible that additional sites are also phosphorylated in a CDK‐dependent manner. PAH1 contains multiple putative target sites containing the minimal consensus sequence S/T‐P and our proteomic analysis provided incomplete coverage (Figure S1). Nevertheless, S162 is so far the only S/T‐P site to have been identified by *in vivo* proteomic studies (Durek *et al*., 2010). In *S. cerevisiae,* Pah1p is phosphorylated at three sites by Cdc28p and at seven by Pho85p (Choi *et al*., 2011, 2012). Not all sites are of equal importance. Disruption of some individual sites leads to a significant reduction in Pah1p activity and membrane association but the largest effect results from simultaneous disruption of all (Choi *et al*., 2012). Pah1p has been shown to be the target of several other kinases, including protein kinase A (Su *et al*., 2012) and protein kinase C (Su *et al*., 2014). The mammalian Pah1p ortholog lipin‐1 is also phosphorylated by mTORC1 (mechanistic target of rapamycin complex1) (Peterson *et al*., 2011). Data contained in the phosphat 4.0 database (Durek *et al*., 2010) suggests that PAH1 is phosphorylated at non S/T‐P sites and therefore is also likely to be targeted by other kinases. These kinases could regulate PAH activity and localization in response to various stimuli, as has been shown in *S. cerevisiae* (Su *et al*., 2012, 2014). PAH1 is mostly present in the cytosol, both when expressed in *Nicotiana benthamiana* leaves (Eastmond *et al*., 2010) and *S. cerevisiae* (Mietkiewska *et al*., 2011). However, Mietkiewska *et al*. (2011) have shown that in cells of *S. cerevisiae* cultured with oleic acid, PAH1 will also localize in the nucleus. The functional significance of this response is unknown but in mammals the nuclear localization of lipin‐1 is also controlled by phosphorylation (Peterson *et al*., 2011). Although mutant analysis suggests that *PAH2* plays a less important role in *A. thaliana* leaves than *PAH1* (Figure 2), the two genes do clearly overlap in function (Eastmond *et al*., 2010). Interestingly the phosphat 4.0 database contains evidence to show that PAH2 is also phosphorylated at several sites including S524 (Durek *et al*., 2010), which corresponds to the consensus S/T‐P. Hence PAH2 may also be phosphorylated by CDKA;1, and indeed by other kinases.

In *S. cerevisiae* dephosphorylation of Pah1p by the Nem1p–Spo7p protein phosphatase complex on the nuclear‐ER membrane, plays a vital role in activating the enzyme and in allowing it to associate with the membrane (Karanasios *et al*., 2010; Choi *et al*., 2011, 2012). This complex (and its function) is also conserved in animals (Kim *et al*., 2007; Han *et al*., 2012), but it has yet to be identified or characterised in plants. The catalytic subunit Nem1p and its animal ortholog C‐terminal domain nuclear envelope phosphatase 1 (CTDNEP1) are related to the small C‐terminal domain phosphatase 1 (SCP1), which is a protein that dephosphorylates the highly conserved heptad repeats in the C‐terminal domain of RNA polymerase II (Hsin and Manley, 2012). *A. thaliana* contains a family of SCP1‐like proteins but those members that have been characterised to date do not localize to the nuclear envelope (Feng *et al*., 2010). The regulatory subunit Spo7p and its animal ortholog, nuclear envelope phosphatase 1‐regulatory subunit 1 (NEP1‐R1), are very small transmembrane proteins that share little sequence similarity with one another (Han *et al*., 2012) and standard bioinformatics tools do not reveal any obvious homologues in *A. thaliana*. Understanding how PAH1 is dephosphorylated and whether a functionally analogous phosphatase complex to Nem1p–Spo7p is involved will require further work.

In conclusion our data suggest that PAH1 is a direct target for phosphorylation by CDKA;1–cyclin complexes in *A. thaliana* and that this post‐translational modification plays an important physiological role in determining the rate of phospholipid biosynthesis. Given that CDKA;1 is a core cell cycle regulator and is instrumental in controlling both the G1 to S and G2 to M transitions (Nowack *et al*., 2012) it is likely that its phosphorylation of PAH1 helps to coordinate phospholipid biosynthesis with fluctuations in demand for membrane biogenesis that must take place at these key stages in cell cycle progression (Jackowski, 1994; Santos‐Rosa *et al*., 2005). In plants, a detailed description of how phospholipid metabolism changes over the course of the cell cycle is still lacking (Kwok and Wong, 2005). Further work will be required to address this knowledge gap and also to attain a more complete understanding of the underlying mechanisms that couple PAH1, and other key lipid metabolic enzymes, to the core cell cycle machinery.

## Experimental Procedures

### Plant material and growth conditions

The construction of the *Arabidopsis thaliana* transgenic lines *pah1 pah2* (DM)*, pah1 pah2 Pro35S:PAH1‐HA* (DMP)*, cdka;1D* and *cdka;1DE* have been described previously (Dissmeyer *et al*., 2007, 2009; Eastmond *et al*., 2010; Craddock *et al*., 2015). Seeds of *A. thaliana* were surface–sterilised, applied to agar plates containing half‐strength MS salts from Sigma‐Aldrich (http://www.sigmaaldrich.com) and imbibed in the dark for 4 days at 4°C after which the plates were then placed vertically in a growth chamber set to 22°C 16 h light/18°C 8 h dark; PPFD = 250 μmol m^−2^ sec^−1^. After 7 days the seedlings were transplanted to soil and grown under the same conditions as described above. PCR performed on genomic DNA was used to genotype T‐DNA mutants (Dissmeyer *et al*., 2007; Eastmond *et al*., 2010).

### PC radiolabelling and PAH enzyme assays

Radiolabel feeding experiments were carried out using rosette leaves that were cut into 2 mm diameter strips with a razor blade. They were then placed in vials containing 0.5 ml of half‐strength MS salts supplemented with 1 mm [*methyl*‐^14^C]choline chloride. The leaf tissue was vacuum infiltrated for 5 min and, following incubation at 22°C with gentle agitation for up to 3 h, the tissue was blotted dry and frozen in liquid nitrogen. Lipid extraction and analysis of the labelled PC were performed using the methods described by Tasseva *et al*. (2004). PAH assays were performed on total homogenates and immunoprecipitates obtained from rosette leaves using the method described by Craddock *et al*. (2015). The PC content was also directly quantified from total tissue extracts using the enzyme‐coupled fluorometric procedure described previously (Eastmond *et al*., 2010).

### Creation of DNA constructs and transformation

*PAH1* ^*S162A*^ was created from a *PAH1* template in entry vector pDONR207 as previously described in (Craddock *et al*., 2015) using the Quikchange Lightning Site‐Directed Mutagenesis Kit from Agilent Technologies (http://www.agilent.com) and primers 5′‐ATGATTTTCAGGATGATCCTCCTGCGCCAACCTCAGAATATGGAAGTGCT‐3′ and 5′‐AGCACTTCCATATTCTGAGGTTGGCGCAGGAGGATCATCCTGAAAATCAT‐3′. The gene cassette was then cloned into the destination vector pEG101 using the Gateway LR clonase enzyme mix from Invitrogen Ltd (http://www.thermofisher.com). Transformation of the plasmids into *Agrobacterium tumefaciens* strain GV3101 was achieved by heat shock and into Arabidopsis WT and *pah1 pah2* by the floral‐dip method (Clough and Bent, 1998). Herbicide resistance was used to select transformants containing T‐DNA insertions and homozygous lines were crossed into *pah1 pah2 cdka;1D* (TM).

### PAH protein extraction and analysis

Subcellular fractionation, protein extraction, quantification, sodium dodecyl sulfate‐polyacrylamide gel electrophoresis (SDS‐PAGE) and immunoblotting were performed as described previously (Craddock *et al*., 2015) but with the following minor modifications. Phosphoenolpyruvate carboxylase activity was measured in subcellular fractions using the method described by Gregory *et al*. (2009). Detection of phosphorylated PAH1‐HA by immunoblotting was carried out with leaf extracts prepared according to the method of Dissmeyer *et al*. (2007), using an ice cold extraction buffer containing phosphatase and protease inhibitors. Immunoprecipitations were then performed by incubating 1 mg of total leaf protein with 40 μg of α‐HA antibodies and 100 μl of protein A‐Sepharose CL‐4B beads from Sigma‐Aldrich (http://sigmaaldrich.com) (10% slurry, w/v) in a total volume of 0.5 ml. Immune complexes were collected by centrifugation at 1500 ***g*** for 20 sec and were washed repeatedly before being used directly for SDS‐PAGE or pre‐treated with CIP solution at 37°C. In addition to using anti‐HA antibodies, membranes were also probed using anti‐(phosphoserine/phosphothreonine)‐proline (α‐MPM2), anti‐His6 (α‐His), anti‐luminal‐binding protein 2 (α‐BiP2) or anti‐PSTAIRE antibodies from EDM Millipore (http://www.merkmillipore.com), Abcam (http://www.abcam.com), Agrisera (http://www.agrisera.com) and Santa Cruz Biotechnology (http://www.scbt.com), respectively.

### Recombinant PAH1 expression and *in vitro* phosphorylation assays

Heterologous expression of His6‐PAH1 in *E. coli* and affinity purification were performed essentially as previously described (Eastmond *et al*., 2010), but with the following modifications. A pellet of *E. coli* strain ArcticExpress RIL cells from Agilent Technologies (http://www.agilent.com) equivalent to 400 ml of culture was washed once with 20 mm Tris–HCl buffer, pH 8.0 and the cells resuspended in 20 ml of Buffer A: 20 mm sodium phosphate (pH 7.4), 0.5 m NaCl, 20 mm imidazole, 7 mm 2‐mercaptoethanol, 1% (v/v) Triton X‐100, 5% (v/v) glycerol, 1:20 (v/v) Protease Inhibitor Cocktail (Sigma‐Aldrich), and 50 μl Lysozyme (100 mg per ml). Cells were disrupted by six short bursts of sonication of 10 sec with a microtip each followed by intervals of 30 sec for cooling. Centrifugation at 40 000 ***g*** for 30 min at 4°C was used to remove cell debris. A 20 μm filter was used to filter the supernatant (cell lysate) and the sample loaded on a 1 ml Ni_2_‐NTA column (GE Healthcare) followed by a 10 ml wash with Buffer A. His6‐tagged proteins were then eluted from the column in 1 ml fractions with a total of 10 ml of Buffer A containing 500 mm imidazole. SDS‐PAGE was used to analyse the fractions which were then stained with Colloidal Coomassie Brilliant Blue G‐250. Pooled enzyme preparations were dialysed against 20 mm sodium phosphate (pH 7.4) containing 10% (v/v) glycerol and 7 mm *2*‐mercaptoethanol before being stored at **−**80°C.

CDK–cyclin complexes were purified from *A. thaliana* flower buds using p13^Suc1^‐Sepharose beads and used for kinase assays following the protocols described by Dissmeyer and Schnittger (2011). Each reaction contained: 30 μl of washed beads together with 8.5 μl of water, 3.5 μl each of 500 mm Tris–Cl (pH 7.8), 150 mm MgCl_2_, 50 mm EGTA, and 10 mm DTT and 5 μl of His6‐PAH1 (~0.1 mg mL^−1^). The addition of 4.1 μl of water, 0.3 μl of 1 mm Li‐ATP, and 6 μCi of [γ‐^32^P]ATP (220 TBq/mmol) started the reactions, which were then incubated for 60 min at room temperature. To terminate the reactions, the mixtures were spotted onto P81 phosphocellulose paper: the papers were then washed five times with 75 mm phosphoric acid and subjected to scintillation counting. Proteomic analysis of unlabelled reaction products was performed following the procedures previously described in Rajangam *et al*. (2013). In brief, the protein was digested in gel with trypsin, the peptides were resolved using ultra‐performance liquid chromatography (UPLC) and analysed by a Quadrupole Time‐of‐Flight mass spectrometer (Q‐ToF MS). The data were used to interrogate the National Center for Biotechnology Information non‐redundant database using a MASCOT tandem MS/MS search (http://www.matrixscience.com) to identify PAH1 peptides.

### Analysis of leaf growth

A kinematic analysis (De Veylder *et al*., 2001) of the abaxial epidermal cells of the first true leaf leave pair between 6 to 22 days after sowing (DAS) was performed following the method described by (Nelissen *et al*., 2013), except that leaves between 6 and 10 DAS were stained with propidium iodide and images were obtained using confocal microscopy. Older leaves were cleared with 70% (v/v) ethanol and mounted in 100% (v/v) lactic acid. The leaves were imaged using a binocular microscope and abaxial epidermal cells from two positions (Nelissen *et al*., 2013) were imaged with an Axiophot microscope from Zeiss (http://www.zeiss.com) using differential interference contrast optics. Cell outlines were generated by hand and analysed using ImageJ (http://rsbweb.nih.gov/ij/) and the average cell area and estimated number of cells per leaf were calculated by dividing leaf blade area by cell area. The rate of cell division was calculated as the relative rate of increase in cell number over the course of time. To achieve this the logarithmic values of the average cell number were locally fitted with a five‐point quadratic function, of which the first derivative was the cell division rate (Nelissen *et al*., 2013).

### Statistical analysis

The GenStat (2011, 14th edition; ©VSN International Ltd, Hemel Hempstead, UK) statistical system was used to carry out Student's *t*‐tests or one‐way analysis of variance (ANOVA). For ANOVA, following significant (*P* < 0.05) F‐test results, means were compared using the appropriate least significant difference value at the 5% (*P* = 0.05) level of significance, on the corresponding degrees of freedom.

### Accession numbers

Sequence data from this article can be found in the GenBank/EMBL data libraries under the following accession numbers: PAH1, At3g09560; PAH2, At5g42870; CDKA;1, At3g48750.

## Supporting information


**Figure S1.** Proteomic analysis of PAH1 phosphorylated *in vitro*.

**Figure S2.** HeliQuest α‐helix analysis of PAH1 and PAH2 N‐termini.

**Table S1.** Leaf PC content of all genotypes.

**Table S2.** Leaf morphology of selected genotypes.

## References

[tpj13321-bib-0001] Al‐Feel, W. , DeMar, J.C. and Wakil, S.J. (2003) A *Saccharomyces cerevisiae* mutant strain defective in acetyl‐CoA carboxylase arrests at the G2/M phase of the cell cycle. Proc. Natl Acad. Sci. USA 100, 3095–3100. 12626751 10.1073/pnas.0538069100 PMC152252

[tpj13321-bib-0002] Atilla‐Gokcumen, G.E. , Muro, E. , Relat‐Goberna, J. , Sasse, S. , Bedigian, A. , Coughlin, M.L. , Garcia‐Manyes, S. and Eggert, U.S. (2014) Dividing cells regulate their lipid composition and localization. Cell, 156, 428–439. 24462247 10.1016/j.cell.2013.12.015 PMC3909459

[tpj13321-bib-0003] Bach, L. , Gissot, L. , Marion, J. , Tellier, F. , Moreau, P. , Satiat‐Jeunemaître, B. , Palauqui, J.C. , Napier, J.A. and Faure, J.D. (2011) Very‐long‐chain fatty acids are required for cell plate formation during cytokinesis in *Arabidopsis thaliana* . J. Cell Sci. 124, 3223–3234. 21896643 10.1242/jcs.074575

[tpj13321-bib-0004] Bahmanyar, S. , Biggs, R. , Schuh, A.L. , Desai, A. , Müller‐Reichert, T. , Audhya, A. , Dixon, J.E. and Oegema, K. (2014) Spatial control of phospholipid flux restricts endoplasmic reticulum sheet formation to allow nuclear envelope breakdown. Genes Dev. 28, 121–126. 24449268 10.1101/gad.230599.113 PMC3909786

[tpj13321-bib-0005] Carman, G.M. and Henry, S.A. (2007) Phosphatidic acid plays a central role in the transcriptional regulation of glycerophospholipid synthesis in *Saccharomyces cerevisiae* . J. Biol. Chem. 282, 37293–37297. 17981800 10.1074/jbc.R700038200 PMC3565216

[tpj13321-bib-0006] Chauhan, N. , Visram, M. , Cristobal‐Sarramian, A. , Sarkleti, F. and Kohlwein, S.D. (2015) Morphogenesis checkpoint kinase Swe1 is the executor of lipolysis‐dependent cell‐cycle progression. Proc. Natl Acad. Sci. USA 112, E1077–E1085. 25713391 10.1073/pnas.1423175112 PMC4364240

[tpj13321-bib-0007] Choi, H.S. , Su, W.M. , Morgan, J.M. , Han, G.S. , Xu, Z. , Karanasios, E. , Siniossoglou, S. and Carman, G.M. (2011) Phosphorylation of phosphatidate phosphatase regulates its membrane association and physiological functions in *Saccharomyces cerevisiae:* identification of SER(602), THR(723), AND SER(744) as the sites phosphorylated by CDC28 (CDK1)‐encoded cyclin‐dependent kinase. J. Biol. Chem. 286, 1486–1498. 21081492 10.1074/jbc.M110.155598 PMC3020757

[tpj13321-bib-0008] Choi, H.S. , Su, W.M. , Han, G.S. , Plote, D. , Xu, Z. and Carman, G.M. (2012) Pho85p‐Pho80p phosphorylation of yeast Pah1p phosphatidate phosphatase regulates its activity, location, abundance, and function in lipid metabolism. J. Biol. Chem. 287, 11290–11301. 22334681 10.1074/jbc.M112.346023 PMC3322823

[tpj13321-bib-0009] Clough, S.J. and Bent, A.F. (1998) Floral dip: a simplified method for Agrobacterium‐mediated transformation of *Arabidopsis thaliana* . Plant J. 16, 735–743. 10069079 10.1046/j.1365-313x.1998.00343.x

[tpj13321-bib-0010] Cornell, R.B. and Northwood, I.C. (2000) Regulation of CTP: phosphocholine cytidylyltransferase by amphitropism and relocalization. Trends Biochem. Sci. 25, 441–447. 10973058 10.1016/s0968-0004(00)01625-x

[tpj13321-bib-0011] Cornell, R. , Grove, G.L. , Rothblat, G.H. and Horwitz, A.F. (1977) Lipid requirement for cell cycling. The effect of selective inhibition of lipid synthesis. Exp. Cell Res. 109, 299–307. 913494 10.1016/0014-4827(77)90009-x

[tpj13321-bib-0012] Craddock, C.P. , Adams, N. , Bryant, F.M. , Kurup, S. and Eastmond, P.J. (2015) Phosphatidic Acid Phosphohydrolase regulates phosphatidylcholine biosynthesis in Arabidopsis by phosphatidic acid‐mediated activation of CTP: Phosphocholine Cytidylyltransferase activity. Plant Cell, 27, 1251–1264. 25862304 10.1105/tpc.15.00037 PMC4558698

[tpj13321-bib-0013] De Veylder, L. , Beeckman, T. , Beemster, G.T. , Krols, L. , Terras, F. , Landrieu, I. , van der Schueren, E. , Maes, S. , Naudts, M. and Inzé, D. (2001) Functional analysis of cyclin‐dependent kinase inhibitors of Arabidopsis. Plant Cell, 13, 1653–1668. 11449057 10.1105/TPC.010087 PMC139548

[tpj13321-bib-0014] Dissmeyer, N. and Schnittger, A. (2011) Use of phospho‐site substitutions to analyze the biological relevance of phosphorylation events in regulatory networks. Methods Mol. Biol. 779, 93–138. 21837563 10.1007/978-1-61779-264-9_6

[tpj13321-bib-0015] Dissmeyer, N. , Nowack, M.K. , Pusch, S. , Stals, H. , Inzé, D. , Grini, P.E. and Schnittger, A. (2007) T‐loop phosphorylation of Arabidopsis CDKA;1 is required for its function and can be partially substituted by an aspartate residue. Plant Cell, 19, 972–985. 17369369 10.1105/tpc.107.050401 PMC1867360

[tpj13321-bib-0016] Dissmeyer, N. , Weimer, A.K. , Pusch, S. ***et al.*** (2009) Control of cell proliferation, organ growth, and DNA damage response operate independently of dephosphorylation of the Arabidopsis Cdk1 homolog CDKA;1. Plant Cell, 21, 3641–3654. 19948791 10.1105/tpc.109.070417 PMC2798325

[tpj13321-bib-0017] Donkor, J. , Zhang, P. , Wong, S. , O'Loughlin, L. , Dewald, J. , Kok, B.P. , Brindley, D.N. and Reue, K. (2009) A conserved serine residue is required for the phosphatidate phosphatase activity but not the transcriptional coactivator functions of lipin‐1 and lipin‐2. J. Biol. Chem. 284, 29968–29978. 19717560 10.1074/jbc.M109.023663 PMC2785625

[tpj13321-bib-0018] Durek, P. , Schmidt, R. , Heazlewood, J.L. , Jones, A. , Maclean, D. , Nagel, A. , Kersten, B. and Schulze, W.X. (2010) PhosPhAt: the *Arabidopsis thaliana* phosphorylation site database. An update. Nucleic Acids Res. 38, D828–D834. 19880383 10.1093/nar/gkp810 PMC2808987

[tpj13321-bib-0019] Eastmond, P.J. , Quettier, A.‐L. , Kroon, J.T. , Craddock, C. , Adams, N. and Slabas, A.R. (2010) Phosphatidic acid phosphohydrolase 1 and 2 regulate phospholipid synthesis at the endoplasmic reticulum in Arabidopsis. Plant Cell, 22, 2796–2811. 20699392 10.1105/tpc.109.071423 PMC2947160

[tpj13321-bib-0020] Enserink, J.M. and Kolodner, R.D. (2010) An overview of Cdk1‐controlled targets and processes. Cell Div. 5, 11. 20465793 10.1186/1747-1028-5-11 PMC2876151

[tpj13321-bib-0021] Feng, Y. , Kang, J.S. , Kim, S. , Yun, D.J. , Lee, S.Y. , Bahk, J.D. and Koiwa, H. (2010) Arabidopsis SCP1‐like small phosphatases differentially dephosphorylate RNA polymerase II C‐terminal domain. Biochem. Biophys. Res. Commun. 397, 355–360. 20513350 10.1016/j.bbrc.2010.05.130

[tpj13321-bib-0022] Ferreira, P.C. , Hemerly, A.S. , Villarroel, R. , Van Montagu, M. and Inzé, D. (1991) The Arabidopsis functional homolog of the p34cdc2 protein kinase. Plant Cell, 3, 531–540. 1840925 10.1105/tpc.3.5.531 PMC160020

[tpj13321-bib-0023] Finck, B.N. , Gropler, M.C. , Chen, Z. , Leone, T.C. , Croce, M.A. , Harris, T.E. , Lawrence, J.C. Jr and Kelly, D.P. (2006) Lipin 1 is an inducible amplifier of the hepatic PGC‐1alpha/PPARalpha regulatory pathway. Cell Metab. 4, 199–210. 16950137 10.1016/j.cmet.2006.08.005

[tpj13321-bib-0024] Gautier, R. , Douguet, D. , Antonny, B. and Drin, G. (2008) HELIQUEST: a web server to screen sequences with specific α‐helical properties. Bioinformatics, 24, 2101–2102. 18662927 10.1093/bioinformatics/btn392

[tpj13321-bib-0025] Golden, A. , Liu, J. and Cohen‐Fix, O. (2009) Inactivation of the *C. elegans* lipin homolog leads to ER disorganization and to defects in the breakdown and reassembly of the nuclear envelope. J. Cell Sci. 122, 1970–1978. 19494126 10.1242/jcs.044743 PMC2723152

[tpj13321-bib-0026] Gorjánácz, M. and Mattaj, I.W. (2009) Lipin is required for efficient breakdown of the nuclear envelope in *Caenorhabditis elegans* . J. Cell Sci. 122, 1963–1969. 19494125 10.1242/jcs.044750

[tpj13321-bib-0027] Gregory, A.L. , Hurley, B.A. , Tran, H.T. , Valentine, A.J. , She, Y.M. , Knowles, V.L. and Plaxton, W.C. (2009) *In vivo* regulatory phosphorylation of the phosphoenolpyruvate carboxylase AtPPC1 in phosphate‐starved *Arabidopsis thaliana* . Biochem J. 420, 57–65. 19228119 10.1042/BJ20082397 PMC2677216

[tpj13321-bib-0028] Gutierrez, C. (2009) The arabidopsis cell division cycle. Arabidopsis Book, 7, e0120. 22303246 10.1199/tab.0120 PMC3243301

[tpj13321-bib-0029] Han, S. , Bahmanyar, S. , Zhang, P. , Grishin, N. , Oegema, K. , Crooke, R. , Graham, M. , Reue, K. , Dixon, J.E. and Goodman, J.M. (2012) Nuclear envelope phosphatase 1‐regulatory subunit 1 (formerly TMEM188) is the metazoan Spo7p ortholog and functions in the lipin activation pathway. J. Biol. Chem. 287, 3123–3137. 22134922 10.1074/jbc.M111.324350 PMC3283218

[tpj13321-bib-0030] Hsin, J.P. and Manley, J.L. (2012) The RNA polymerase II CTD coordinates transcription and RNA processing. Genes Dev. 26, 2119–2137. 23028141 10.1101/gad.200303.112 PMC3465734

[tpj13321-bib-0031] Inzé, D. and De Veylder, L. (2006) Cell cycle regulation in plant development. Annu. Rev. Genet. 40, 77–105. 17094738 10.1146/annurev.genet.40.110405.090431

[tpj13321-bib-0032] Ito, J. , Taylor, N.L. , Castleden, I. , Weckwerth, W. , Millar, A.H. and Heazlewood, J.L. (2009) A survey of the *Arabidopsis thaliana* mitochondrial phosphoproteome. Proteomics, 9, 4229–4240. 19688752 10.1002/pmic.200900064

[tpj13321-bib-0033] Jackowski, S. (1994) Coordination of membrane phospholipid synthesis with the cell cycle. J. Biol. Chem. 269, 3858–3867. 8106431

[tpj13321-bib-0034] Jackowski, S. (1996) Cell cycle regulation of membrane phospholipid metabolism. J. Biol. Chem. 271, 20219–20222. 8702749 10.1074/jbc.271.34.20219

[tpj13321-bib-0035] Jackowski, S. and Fagone, P. (2005) CTP: phosphocholine cytidylyltransferase: paving the way from gene to membrane. J. Biol. Chem. 280, 853–856. 15536089 10.1074/jbc.R400031200

[tpj13321-bib-0036] Janero, D.R. and Barrnett, R. (1981) Thylakoid membrane biogenesis in *Chlamydomonas reinhardtii* 137+: cell cycle variations in the synthesis and assembly of polar glycerolipid. J. Cell Biol. 91, 126–134. 7298713 10.1083/jcb.91.1.126 PMC2111926

[tpj13321-bib-0037] Jones, A.M. , MacLean, D. , Studholme, D.J. , Serna‐Sanz, A. , Andreasson, E. , Rathjen, J.P. and Peck, S.C. (2009) Phosphoproteomic analysis of nuclei‐enriched fractions from *Arabidopsis thaliana* . J. Proteomics. 72, 439–451. 19245862 10.1016/j.jprot.2009.02.004

[tpj13321-bib-0038] Joseleau‐Petit, D. , Kepes, F. and Kepes, A. (1984) Cyclic changes of the rate of phospholipid synthesis during synchronous growth of *Escherichia coli* . Eur. J. Biochem. 139, 605–611. 6365557 10.1111/j.1432-1033.1984.tb08047.x

[tpj13321-bib-0039] Karanasios, E. , Han, G.S. , Xu, Z. , Carman, G.M. and Siniossoglou, S. (2010) A phosphorylation‐regulated amphipathic helix controls the membrane translocation and function of the yeast phosphatidate phosphatase. Proc. Natl Acad. Sci. USA 107, 17539–17544. 20876142 10.1073/pnas.1007974107 PMC2955120

[tpj13321-bib-0040] Keogh, M.R. , Courtney, P.D. , Kinney, A.J. and Dewey, R.E. (2009) Functional characterization of phospholipid N‐methyltransferases from Arabidopsis and soybean. J. Biol. Chem. 284, 15439–15447. 19366698 10.1074/jbc.M109.005991 PMC2708841

[tpj13321-bib-0041] Kim, Y. , Gentry, M.S. , Harris, T.E. , Wiley, S.E. , Lawrence, J.C. Jr and Dixon, J.E. (2007) A conserved phosphatase cascade that regulates nuclear membrane biogenesis. Proc. Natl Acad. Sci. USA 104, 6596–6601. 17420445 10.1073/pnas.0702099104 PMC1871831

[tpj13321-bib-0042] Kinney, A.J. , Clarkson, D.T. and Loughman, B.C. (1987) The regulation of phosphatidylcholine biosynthesis in rye (*Secale cereale*) roots. Biochem. J. 242, 755–759. 3036101 10.1042/bj2420755 PMC1147775

[tpj13321-bib-0043] Knacker, T. , Harwood, J.L. , Hunter, C.N. and Russell, N.J. (1985) Lipid biosynthesis in synchronized cultures of the photosynthetic bacterium *Rhodopseudomonas sphaeroides* . Biochem. J. 229, 701–710. 3902003 10.1042/bj2290701 PMC1145114

[tpj13321-bib-0044] Koeberle, A. , Shindou, H. , Koeberle, S.C. , Laufer, S.A. , Shimizu, T. and Werz, O. (2013) Arachidonoyl‐phosphatidylcholine oscillates during the cell cycle and counteracts proliferation by suppressing Akt membrane binding. Proc. Natl Acad. Sci. USA 110, 2546–2551. 23359699 10.1073/pnas.1216182110 PMC3574958

[tpj13321-bib-0045] Kwok, A.C. and Wong, J.T. (2005) Lipid biosynthesis and its coordination with cell cycle progression. Plant Cell Physiol. 46, 1973–1986. 16239308 10.1093/pcp/pci213

[tpj13321-bib-0046] Loewen, C.J. , Gaspar, M.L. , Jesch, S.A. , Delon, C. , Ktistakis, N.T. , Henry, S.A. and Levine, T.P. (2004) Phospholipid metabolism regulated by a transcription factor sensing phosphatidic acid. Science, 304, 1644–1647. 15192221 10.1126/science.1096083

[tpj13321-bib-0047] Makarova, M. , Gu, Y. , Chen, J.S. , Beckley, J.R. , Gould, K.L. and Oliferenko, S. (2016) Temporal regulation of lipin activity diverged to account for differences in mitotic programs. Curr. Biol. 26, 237–243. 26774782 10.1016/j.cub.2015.11.061 PMC4728079

[tpj13321-bib-0048] Mall, M. , Walter, T. , Gorjánácz, M. , Davidson, I.F. , Nga Ly‐Hartig, T.B. , Ellenberg, J. and Mattaj, I.W. (2012) Mitotic lamin disassembly is triggered by lipid‐mediated signaling. J. Cell Biol. 198, 981–990. 22986494 10.1083/jcb.201205103 PMC3444782

[tpj13321-bib-0049] Martinez, M.C. , Jørgensen, J.E. , Lawton, M.A. , Lamb, C.J. and Doerner, P.W. (1992) Spatial pattern of cdc2 expression in relation to meristem activity and cell proliferation during plant development. Proc. Natl Acad. Sci. USA 89, 7360–7364. 1502145 10.1073/pnas.89.16.7360 PMC49709

[tpj13321-bib-0051] Mietkiewska, E. , Siloto, R.M. , Dewald, J. , Shah, S. , Brindley, D.N. and Weselake, R.J. (2011) Lipins from plants are phosphatidate phosphatases that restore lipid synthesis in a pah1Δ mutant strain of *Saccharomyces cerevisiae* . FEBS J. 278, 764–775. 21205207 10.1111/j.1742-4658.2010.07995.x

[tpj13321-bib-0052] Nakagami, H. , Sugiyama, N. , Mochida, K. , Daudi, A. , Yoshida, Y. , Toyoda, T. , Tomita, M. , Ishihama, Y. and Shirasu, K. (2010) Large‐scale comparative phosphoproteomics identifies conserved phosphorylation sites in plants. Plant Physiol. 153, 1161–1174. 20466843 10.1104/pp.110.157347 PMC2899915

[tpj13321-bib-0053] Nakamura, Y. , Koizumi, R. , Shui, G. , Shimojima, M. , Wenk, M.R. , Ito, T. and Ohta, H. (2009) Arabidopsis lipins mediate eukaryotic pathway of lipid metabolism and cope critically with phosphate starvation. Proc. Natl Acad. Sci. USA 106, 20978–20983. 19923426 10.1073/pnas.0907173106 PMC2791602

[tpj13321-bib-0054] Nelissen, H. , Rymen, B. , Coppens, F. , Dhondt, S. , Fiorani, F. and Beemster, G.T. (2013) Kinematic analysis of cell division in leaves of mono‐ and dicotyledonous species: a basis for understanding growth and developing refined molecular sampling strategies. Methods Mol. Biol. 959, 247–264. 23299681 10.1007/978-1-62703-221-6_17

[tpj13321-bib-0055] Nowack, M.K. , Harashima, H. , Dissmeyer, N. , Zhao, X. , Bouyer, D. , Weimer, A.K. , De Winter, F. , Yang, F. and Schnittger, A. (2012) Genetic framework of cyclin‐dependent kinase function in Arabidopsis. Dev. Cell 22, 1030–1040. 22595674 10.1016/j.devcel.2012.02.015

[tpj13321-bib-0056] Ohlrogge, J. and Browse, J. (1995) Lipid biosynthesis. Plant Cell, 7, 957–970. 7640528 10.1105/tpc.7.7.957 PMC160893

[tpj13321-bib-0057] Peterson, T.R. , Sengupta, S.S. , Harris, T.E. ***et al.*** (2011) mTOR complex 1 regulates lipin 1 localization to control the SREBP pathway. Cell, 146, 408–420. 21816276 10.1016/j.cell.2011.06.034 PMC3336367

[tpj13321-bib-0058] Polyn, S. , Willems, A. and De Veylder, L. (2015) Cell cycle entry, maintenance, and exit during plant development Curr. Opin. Plant Biol. 23, 1–7. 10.1016/j.pbi.2014.09.012 25449720

[tpj13321-bib-0505] Rajangam, A.S. , Gidda, S.K. , Craddock, C. , Mullen, R.T. , Dyer, J.M. and Eastmond, P.J. (2013) Molecular characterization of the fatty alcohol oxidase pathway for wax‐ester mobilization in germinated jojoba seeds. Plant Physiol., 161, 72–80. 23166353 10.1104/pp.112.208264 PMC3532287

[tpj13321-bib-0059] Reiland, S. , Finazzi, G. , Endler, A. ***et al.*** (2011) Comparative phosphoproteome profiling reveals a function of the STN8 kinase in fine‐tuning of cyclic electron flow (CEF). Proc. Natl Acad. Sci. USA 108, 12955–12960. 21768351 10.1073/pnas.1104734108 PMC3150903

[tpj13321-bib-0060] Saitoh, S. , Takahashi, K. , Nabeshima, K. , Yamashita, Y. , Nakaseko, Y. , Hirata, A. and Yanagida, M. (1996) Aberrant mitosis in fission yeast mutants defective in fatty acid synthetase and acetyl CoA carboxylase. J. Cell Biol. 134, 949–961. 8769419 10.1083/jcb.134.4.949 PMC2120970

[tpj13321-bib-0061] Santos‐Rosa, H. , Leung, J. , Grimsey, N. , Peak‐Chew, S. and Siniossoglou, S. (2005) The yeast lipin Smp2 couples phospholipid biosynthesis to nuclear membrane growth. EMBO J. 24, 1931–1941. 15889145 10.1038/sj.emboj.7600672 PMC1142606

[tpj13321-bib-0062] Segers, G. , Gadisseur, I. , Bergounioux, C. , de Almeida Engler, J. , Jacqmard, A. , Van Montagu, M. and Inzé, D. (1996) The Arabidopsis cyclin‐dependent kinase gene cdc2bAt is preferentially expressed during S and G2 phases of the cell cycle. Plant J. 10, 601–612. 8893539 10.1046/j.1365-313x.1996.10040601.x

[tpj13321-bib-0063] Su, W.M. , Han, G.S. , Casciano, J. and Carman, G.M. (2012) Protein kinase A‐mediated phosphorylation of Pah1p phosphatidate phosphatase functions in conjunction with the Pho85p‐Pho80p and Cdc28p‐cyclin B kinases to regulate lipid synthesis in yeast. J. Biol. Chem. 287, 33364–33376. 22865862 10.1074/jbc.M112.402339 PMC3460439

[tpj13321-bib-0064] Su, W.M. , Han, G.S. and Carman, G.M. (2014) Cross‐talk phosphorylations by protein kinase C and Pho85p‐Pho80p protein kinase regulate Pah1p phosphatidate phosphatase abundance in *Saccharomyces cerevisiae* . J. Biol. Chem. 289, 18818–18830. 24876385 10.1074/jbc.M114.581462 PMC4081924

[tpj13321-bib-0065] Szymanski, J. , Brotman, Y. , Willmitzer, L. and Cuadros‐Inostroza, Á. (2014) Linking gene expression and membrane lipid composition of Arabidopsis. Plant Cell, 26, 915–928. 24642935 10.1105/tpc.113.118919 PMC4001401

[tpj13321-bib-0066] Tasseva, G. , Richard, L. and Zachowski, A. (2004) Regulation of phosphatidylcholine biosynthesis under salt stress involves choline kinases in *Arabidopsis thaliana* . FEBS Lett. 566, 115–120. 15147879 10.1016/j.febslet.2004.04.015

[tpj13321-bib-0067] Tercé, F. , Brun, H. and Vance, D.E. (1994) Requirement of phosphatidylcholine for normal progression through the cell cycle in C3H/10T1/2 fibroblasts. J. Lipid Res. 35, 2130–2142. 7897311

[tpj13321-bib-0068] Ugrankar, R. , Liu, Y. , Provaznik, J. , Schmitt, S. and Lehmann, M. (2011) Lipin is a central regulator of adipose tissue development and function in *Drosophila melanogaster* . Mol. Cell. Biol. 31, 1646–1656. 21300783 10.1128/MCB.01335-10 PMC3126333

[tpj13321-bib-0069] Wang, P. , Xue, L. , Batelli, G. , Lee, S. , Hou, Y.J. , Van Oosten, M.J. , Zhang, H. , Tao, W.A. and Zhu, J.K. (2013) Quantitative phosphoproteomics identifies SnRK2 protein kinase substrates and reveals the effectors of abscisic acid action. Proc. Natl Acad. Sci. USA 110, 11205–11210. 23776212 10.1073/pnas.1308974110 PMC3703982

[tpj13321-bib-0070] Wang, L. , Kazachkov, M. , Shen, W. , Bai, M. , Wu, H. and Zou, J. (2014) Deciphering the roles of Arabidopsis LPCAT and PAH in phosphatidylcholine homeostasis and pathway coordination for chloroplast lipid synthesis. Plant J. 80, 965–976. 25268378 10.1111/tpj.12683

[tpj13321-bib-0071] Zhang, H. , Zhou, H. , Berke, L. , Heck, A.J. , Mohammed, S. , Scheres, B. and Menke, F.L. (2013) Quantitative phosphoproteomics after auxin‐stimulated lateral root induction identifies an SNX1 protein phosphorylation site required for growth. Mol. Cell Proteomics 12, 1158–1169. 23328941 10.1074/mcp.M112.021220 PMC3650328

