# Supplementary material for: Cyclin‐dependent kinase activity enhances phosphatidylcholine biosynthesis in Arabidopsis by repressing phosphatidic acid phosphohydrolase activity
Source: Plant J. 2016 Dec 1;89(1):3–14. doi: 10.1111/tpj.13321 (PMC5299491; doi:10.1111/tpj.13321)
Supplement: Supplementary file 1 — Figure S1. Proteomic analysis of PAH1 phosphorylated in vitro. [file TPJ-89-3-s001.pdf]

**(a)**

MGSSHHHHHHSSGLVPRGSHMASMSLVGRVGSLISQGVYSVATPFHPFGGAIDVIVVQQQDGSFRSTPWYVRFGK
FQGVLKGAEKFVRISVNGTEADFHMYLDNSGEAYFIREVDPAANDTNNLISGSENNNGNQNNGVTYRLEHSLSDS
GTGELREGFDPLSRLERTESDCNRRFYDFQDDPPSPTSEYGSARFDNLNVESYGDSQGSDSEVVLVSIDGHILTA
PVSVAEQEAENLRLNTPQFHLAPGDGTEFCEGNTEFASSETPWDTEYIDKVEESSDTANIASDKVDAINDERNDL
DSHSRDNAEKDSHDAERDLLGSCLEQSELTKTSENVKSEEPGPTFEDRNLKEGEFPLRTIMENDRSEDEVTIESI
DTLVDSFESSTTQITIEEVKTTEGSRISVDSNADSECKDEQTSAETAILFNNQESSISVDSNADSECKDEQPRIS
AETAILINNQEGGIIESEDQDSERVSIDSTREEVDKDNEDRKTVVSVGVTSSVDEGEPDTDQRYELSLCKDELRQ
GMGLSAAAEVFDAHMISKEEYINSATSILESENLVVRIRETYMPWTKAARIVLGKAVFDLDLDIQPDDVISVEEN
ESPKPKDDETTITPSSSGTRWRLWPIPFRRVKTVEHTGSNSSSEEDLFVDSEPGLQNSPETQSTTESRHESPRRQ
LVRTNVPTNEQIASLNLKDGQNMITFSFSTRVLGTQQVDAHIYRWRWDTKIVISDVDGTITKSDVLGQFMPFIGK
DWTQSGVAKLFSAIKENGYQLLFLSARAIVQAYLTRNFLNNLKQDGKALPTGPVVISPDGLFPALYREVIRRAPH
EFKIACLEDIRKLFPTDYNPFYAGFGNRDTDELSYRKLGIPKGKIFIINPKGEVATGHRIDVKKSYTSLHTLVND
MFPPTSLVEQEDYNPWNFWKLPIEEVE

**(b)**

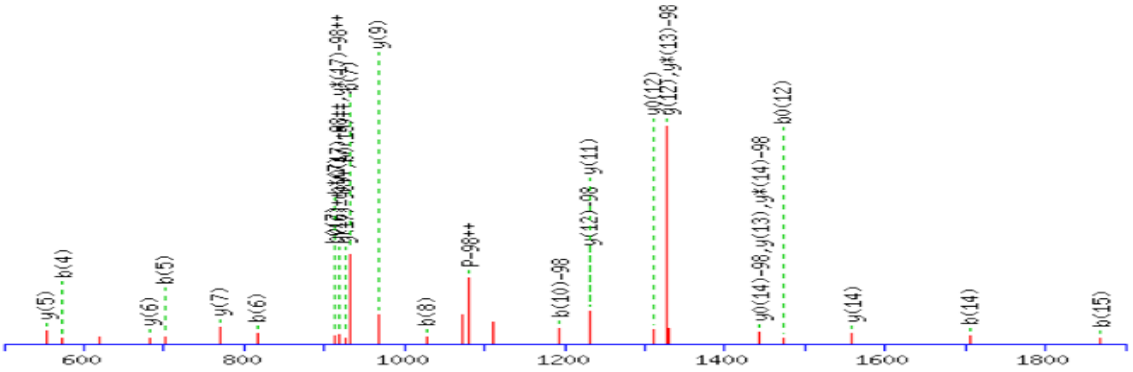


**Figure S1.** Proteomic analysis of PAH1 phosphorylated *in vitro* by purified CDK-cyclin complexes. (a) Sequence of His6-PAH1 showing 33 tryptic peptides (red/green) indentified by LC-MS/MS, following a Mascot MS/MS Ions Search (P < 0.05). Coverage = 43%. His6 tag sequence in blue. (b) MS/MS fragmentation of peptide FYDFQDDPP[pS]PTSEYGSAR (underlined in A) identified following *in vitro* CDK-dependent phosphorylation. Calculated mass = 2257.8841, ion score = 63, matched b ions: b(4), b(5), b(6), b(7), b(8), b(10)-98, b(14), b(15), matched y ions: y(5), y(6), y(7), y(9), y(11), y(12)-98, y(12), y(13), y(14), y(16)++, y(17)-98++, precursor origin neutral loss: +.
