# Supplementary material for: Cyclin‐dependent kinase activity enhances phosphatidylcholine biosynthesis in Arabidopsis by repressing phosphatidic acid phosphohydrolase activity
Source: Plant J. 2016 Dec 1;89(1):3–14. doi: 10.1111/tpj.13321 (PMC5299491; doi:10.1111/tpj.13321)
Supplement: Supplementary file 2 — Figure S2. HeliQuest α‐helix analysis of PAH1 and PAH2 N‐termini. [file TPJ-89-3-s002.pdf]

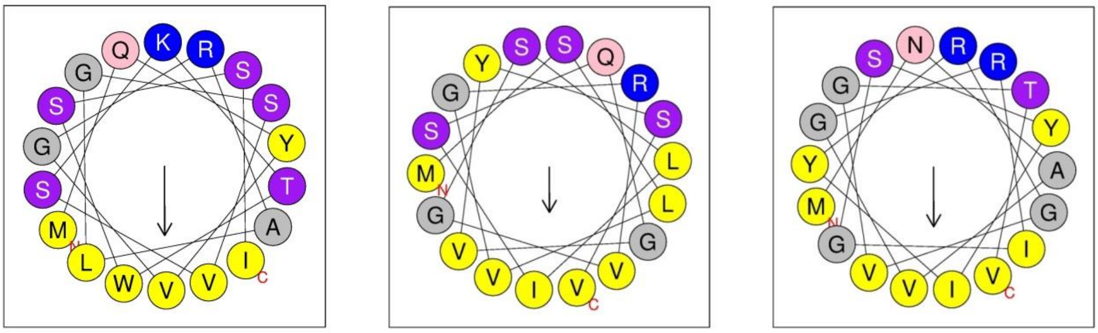

Figure S2. HeliQuest α-helix analysis (http://heliquest.ipmc.cnrs.fr/) of N-terminal 18 aa for yeast Pah1p and Arabidopsis PAH1 and PAH2 (left to right). Hydrophobicity = 0.476, 0.604 & 0.462; hydrophobic moment = 0.557, 0.365 & 0.482 µH; net charge = 2, 1 & 2 z; hydrophobic face = AIVVWLM, LLGVVIVVGM & YAGIVIVVGMY. N- and C-termini highlighted in red. Nonpolar residues in yellow.
