# Supplementary material for: Cyclin‐dependent kinase activity enhances phosphatidylcholine biosynthesis in Arabidopsis by repressing phosphatidic acid phosphohydrolase activity
Source: Plant J. 2016 Dec 1;89(1):3–14. doi: 10.1111/tpj.13321 (PMC5299491; doi:10.1111/tpj.13321)
Supplement: Supplementary file 3 — Table S1. Leaf PC content of all genotypes. [file TPJ-89-3-s003.pdf]

Table S1. Leaf PC content of all genotypes.

Genotype	PC content (relative units)
WT *(wild type)*	1.00 ±0.11
cdkA;1D (cdka;1 ProCDKA;1:CDKA;1^{T161D})	0.76 ±0.09*
cdkA;1DE (cdka;1 ProCDKA;1:CDKA;1$^{T14D-Y15E}$)	0.72 ±0.07*
DM *(pah1 pah2)*	1.89 ±0.16*
DMP1 *(pah1 pah2 Pro35S:PAH1-HA)*	0.97 ±0.09
DMP2 *(pah1 pah2 Pro35S:PAH1-HA)*	0.96 ±0.07
DMp1 *(pah1 pah2 Pro35S:PAH1^{S162A}-HA)*	0.81 ±0.04*
DMp2 *(pah1 pah2 Pro35S:PAH1^{S162A}-HA)*	0.79 ±0.06*
TM *(pah1 pah2 cdkA;1D)*	1.82 ±0.14*
TMP *(pah1 pah2 cdkA;1D Pro35S:PAH1-HA)*	0.73 ±0.08*
TMp *(pah1 pah2 cdkA;1D Pro35S:PAH1^{S162A}-HA)*	0.73 ±0.10*

PC content was measured on a per unit fresh weight basis and values are the mean ± SE from four separate pools of leaf material from each genotype. Asterisks denote a statistically significant difference from WT (P < 0.05).
