# Supplementary material for: Cyclin‐dependent kinase activity enhances phosphatidylcholine biosynthesis in Arabidopsis by repressing phosphatidic acid phosphohydrolase activity
Source: Plant J. 2016 Dec 1;89(1):3–14. doi: 10.1111/tpj.13321 (PMC5299491; doi:10.1111/tpj.13321)
Supplement: Supplementary file 4 — Table S2. Leaf morphology of selected genotypes. [file TPJ-89-3-s004.pdf]

Table S2. Leaf morphology of selected genotypes.

Genotype	Leaf area (mm^2)	Cell number	Cell area (μm^2)
cdkA;1D	7.1 ±0.1	4304 ±120	1650 ±25
TM	6.8 ±0.2	4099 ±171	1659 ±39

Values are the mean ± SE of measurements on the abaxial surfaces of mature leaves from three plants of each genotype.
