# Supplementary material for: Cyclin‐dependent kinase activity enhances phosphatidylcholine biosynthesis in Arabidopsis by repressing phosphatidic acid phosphohydrolase activity
Source: Plant J. 2016 Dec 1;89(1):3–14. doi: 10.1111/tpj.13321 (PMC5299491; doi:10.1111/tpj.13321)
Supplement: Supplementary file 5 [file TPJ-89-3-s005.docx]

**SUPPORTING INFORMATION LEGENDS**

**Figure S1.**  Proteomic analysis of PAH1 phosphorylated *in vitro* by purified CDK-cyclin complexes. (a) Sequence of His6-PAH1 showing 33 tryptic peptides (red/green) indentified by LC-MS/MS, following a Mascot MS/MS Ions Search (P < 0.05). Coverage = 43%. His6 tag sequence in blue. (b) MS/MS fragmentation of peptide FYDFQDDPP[pS]PTSEYGSAR (underlined in A) identified following *in vitro* CDK-dependent phosphorylation. Calculated mass = 2257.8841, ion score = 63, matched b ions: b(4), b(5), b(6), b(7), b(8), b(10)-98, b(14), b(15), matched y ions: y(5), y(6), y(7), y(9), y(11), y(12)-98, y(12), y(13), y(14), y(16)++, y(17)-98++, precursor origin neutral loss: +.

**Figure S2.** HeliQuest α-helix analysis (http://heliquest.ipmc.cnrs.fr/) of N-terminal 18 aa for yeast Pah1p and Arabidopsis PAH1 and PAH2 (left to right). Hydrophobicity = 0.476, 0.604 & 0.462; hydrophobic moment = 0.557, 0.365 & 0.482 μH; net charge = 2, 1 & 2 z; hydrophobic face = AIVVWLM, LLGVVIVVGM & YAGIVIVVGMY. N- and C-termini highlighted in red. Nonpolar residues in yellow.

**Table S1.** Leaf PC content of all genotypes.

**Table S2.** Leaf morphology of selected genotypes.
